# VASP, zyxin and TES are tension-dependent members of Focal Adherens Junctions independent of the α-catenin-vinculin module

**DOI:** 10.1038/srep17225

**Published:** 2015-11-27

**Authors:** Joppe Oldenburg, Gerard van der Krogt, Floor Twiss, Annika Bongaarts, Yasmin Habani, Johan A. Slotman, Adriaan Houtsmuller, Stephan Huveneers, Johan de Rooij

**Affiliations:** 1 Dept. Molecular Cancer Research, Center for Molecular Medicine, University Medical Center Utrecht, Stratenum 3.231, Universiteitsweg 100, 3584 CG, Utrecht, the Netherlands; 2 Erasmus Optical Imaging Center, Department of Pathology, Erasmus MC, Dr. Molewaterplein 50-60, 3015 GE Rotterdam, the Netherlands; 3 Department of Molecular Cell Biology, Sanquin Research and Swammerdam Institute for Life Sciences, University of Amsterdam. Plesmanlaan 125, 1066 CX, Amsterdam, the Netherlands

## Abstract

Mechanical forces are integrated at cadherin-based adhesion complexes to regulate morphology and strength of cell-cell junctions and organization of associated F-actin. A central mechanosensor at the cadherin complex is α-catenin, whose stretching recruits vinculin to regulate adhesion strength. The identity of the F-actin regulating signals that are also activated by mechanical forces at cadherin-based junctions has remained elusive. Here we identify the actin-regulators VASP, zyxin and TES as members of punctate, tensile cadherin-based junctions called Focal Adherens Junctions (FAJ) and show that they display mechanosensitive recruitment similar to that of vinculin. However, this recruitment is not altered by destroying or over-activating the α-catenin/vinculin module. Structured Illumination Microscopy (SIM) indicates that these tension sensitive proteins concentrate at locations within FAJs that are distinct from the core cadherin complex proteins. Furthermore, localization studies using mutated versions of VASP and zyxin indicate that these two proteins require binding to each other in order to localize to the FAJs. We conclude that there are multiple force sensitive modules present at the FAJ that are activated at distinct locations along the cadherin-F-actin axis and regulate specific aspects of junction dynamics.

The development and integrity of multicellular tissues is regulated by chemical and mechanical cues from the extracellular environment. Sites of adhesion between cells and the extracellular matrix (ECM), mediated predominantly by integrins, are the primary sensors of extracellular mechanics^1^. They orchestrate cellular responses that include adaptation of the mechanics of the intracellular cytoskeletal networks. In other words, cellular cytoskeletons stiffen with increasing stiffness of their environment^2^,^3^. Such cytoskeletal stiffening responses are then further relayed to regulate a diversity of processes that include morphogenetic movements^4^ as well as stem cell maintenance or differentiation^5^.

One of the protein complexes that emerges as a key responder to changes in cytoskeletal stiffness is the classical cadherin complex that mediates cell-cell adhesion in all soft tissues^6^,^7^,^8^. The cadherin complex forms a direct interaction with the actomyosin cytoskeleton and changes in cytoskeletal organization and increases in actomyosin contractility result in fluctuations of tension across the cadherin-catenin-F-actin axis. The cadherin complex directly responds to increased tension by activating feedback signals that results in both actomyosin remodeling as well as adhesion remodeling^9^. A key tension sensor in this feedback is α-catenin, which becomes stretched under tension to open up its vinculin binding site. Subsequent vinculin recruitment results in adhesion tightening that protects junctions from breaking^6^,^9^,^10^,^11^. Once recruited, vinculin itself will also become part of the force-chain at the cadherin-actomyosin interface, which may lead to additional mechanosensitive events. One of the interactors of vinculin, VASP was recently found to mediate vinculin-dependent actin remodeling at the apical Zonula Adherens (ZA) in columnar epithelial cells^12^. These recent studies emphasize that the cadherin complex is not static intercellular glue, but that it is a dynamic, tension regulated complex whose composition determines the organization and stability of multicellular tissues.

The dynamics of the cadherin adhesion complex are uniquely visible during the rapid remodeling of cell-cell junctions that occur in endothelial monolayers and other flat epithelia that are stimulated by morphogenetic signals such as thrombin, VEGF and HGF. During these processes, cytoskeletal rearrangements occur and we have recently shown how this results in the formation of a special type of cell-cell contact that we called Focal Adherens Junction (FAJ)^9^. As opposed to other cell-cell contacts (linear adherens junctions (LAJ) in flat epithelia and zonula adherens junctions (ZAJ) in columnar epithelia) that are associated with parallel oriented F-actin bundles, FAJs are attached to radially-oriented F-actin bundles that confer tension to them^13^. The tension dependent recruitment of vinculin to FAJs and the feedback towards adhesion strengthening is crucial to protect junctions from breaking during these remodeling processes. An unexpected observation was that, in the absence of vinculin recruitment, LAJs still remodel into FAJs, including reorganization of the associated F-actin structures^9^. Remaining questions include how the different types of adhesion (FAJs, LAJs, and ZAJs) relate to each other; How the interaction with F-actin is controlled during adhesion remodeling; How the structure of F-actin is organized by the cadherin complex and whether and how additional tension-sensitive events occur that control distant processes like intracellular signaling and transcriptional control?

In the current study, we set out to investigate proteins possibly involved in the regulation of F-actin organization at FAJs. VASP is a vinculin interactor that possesses actin binding and (anti-) capping activities, it is involved in the formation of actin-bundles that drive fillopodial protrusion and also has a function in actin filament elongation^14^,^15^,^16^. VASP is present in cell-cell junctions that form between sparsely grown keratinocytes and in zonula adherens junctions in breast cancer cells^12^,^17^. Zyxin is a VASP interactor that binds to the same EVH1 domain as vinculin does. Zyxin has previously been implicated in mechanoresponses at integrin-based focal adhesions (FA)^18^ as well as cadherin based cell-cell contacts^19^,^20^,^21^. Zyxin and VASP were both found to concentrate at FAJs in MDCK cells stimulated by HGF^19^. Zyxin was shown to be recruited to tensile sites in the actin cytoskeleton through its LIM domains^22^,^23^. Zyxin-dependent recruitment of VASP to sites of tension-induced cytoskeletal damage was found to regulate actin filament repair^24^. Testin (TES) is a zyxin-related LIM-domain protein with tumor suppressive activity that interacts with zyxin and VASP family proteins at integrin-based FAs^25^. We show here that VASP, zyxin and TES are all tension sensitive members of the cadherin junction. Mutational analyses indicate that VASP and zyxin require binding to each other in order to localize to the FAJs. Moreover, this localization is independent of the α-catenin/vinculin module. Superresolution microscopy indicates that these actin regulators concentrate at domains distinct from α-catenin, which thus represent novel mechanosensitive systems at the cadherin-F-actin interface.

## Results

### Actin organization at FAJs is not perturbed by vinculin absence

**Figure 1:**
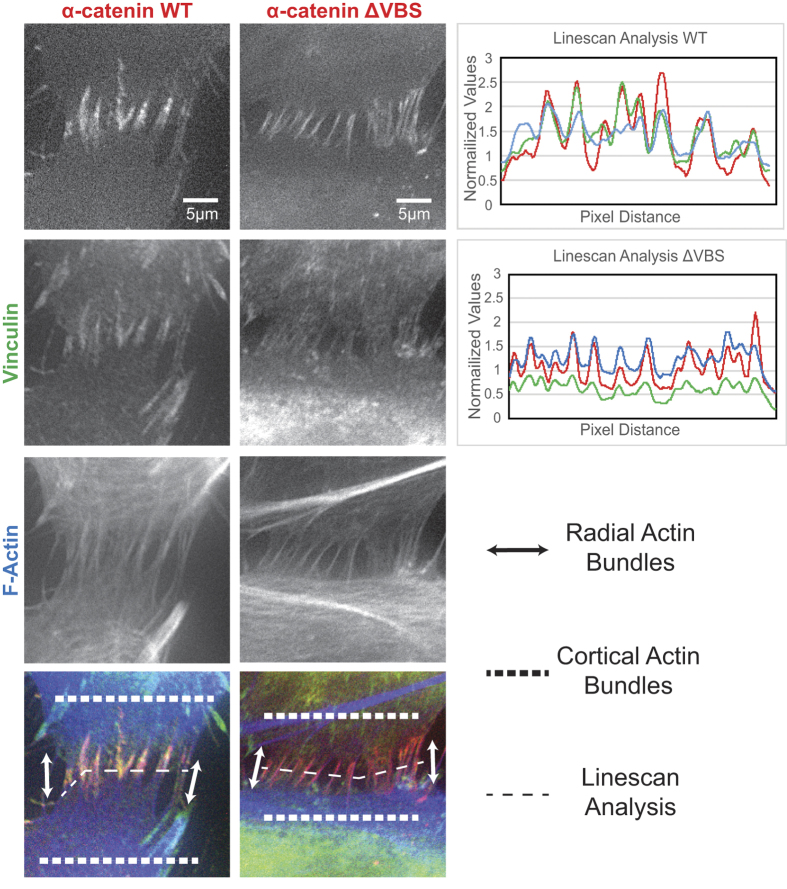
Similar actin organization at FAJs with and without α-catenin-vinculin module. Human Umbilical Vein Endothelial Cells (HUVEC) were treated with shRNA to silence α-catenin, and subsequently rescued with either α-catenin WT or α-catenin ΔVBS (eGFP). Cells were fixed and IF labelled for vinculin and F-actin, and imaged by wide-field microscopy. Zoom in images demonstrate FAJs with their specific actin organization of radial actin bundles and cortical actin bundles. The α-catenin ΔVBS FAJs show a lack of vinculin, which was confirmed by line scan analysis of the pixel intensity in different fluorescent channels.

Focal Adherens Junctions (FAJs) are characterized by a punctate appearance, contact to radial F-actin fibers, the presence of vinculin and increased tension^9^. To investigate whether the presence of vinculin is necessary for the bundling of radial actin fibers at FAJs, we silenced endogenous α-catenin in Human Umbilical Vein Endothelial Cells (HUVEC) and rescued cell-cell junction formation by overexpressing murine, eGFP-tagged α-catenin-WT or α-catenin-ΔVBS (a vinculin-binding deficient mutant previously characterized in^9^,^26^). A similar experiment was previously shown by us in Huveneers *et al.*^9^ to proof that HUVECs expressing α-catenin ΔVBS demonstrate a strong reduction of vinculin at the FAJs. Importantly, we also noticed that the radial actin bundles at FAJs are very similar in α-catenin ΔVBS and α-catenin-WT HUVECs (Fig. 1). Radial actin bundles run towards the cell-cell contacts and a marked concentration of F-actin occurs at the FAJ. Line scan analyses (Fig. 1, right panel) confirm the perturbation of vinculin recruitment to FAJs in α-catenin-ΔVBS cells, while the actin signal concentrates with that of α-catenin to a similar extent as in α-catenin-WT cells. This indicates that vinculin is not necessary for the specific actin organization present at FAJs.

### VASP, zyxin and TES are localized at FAJs

**Figure 2:**
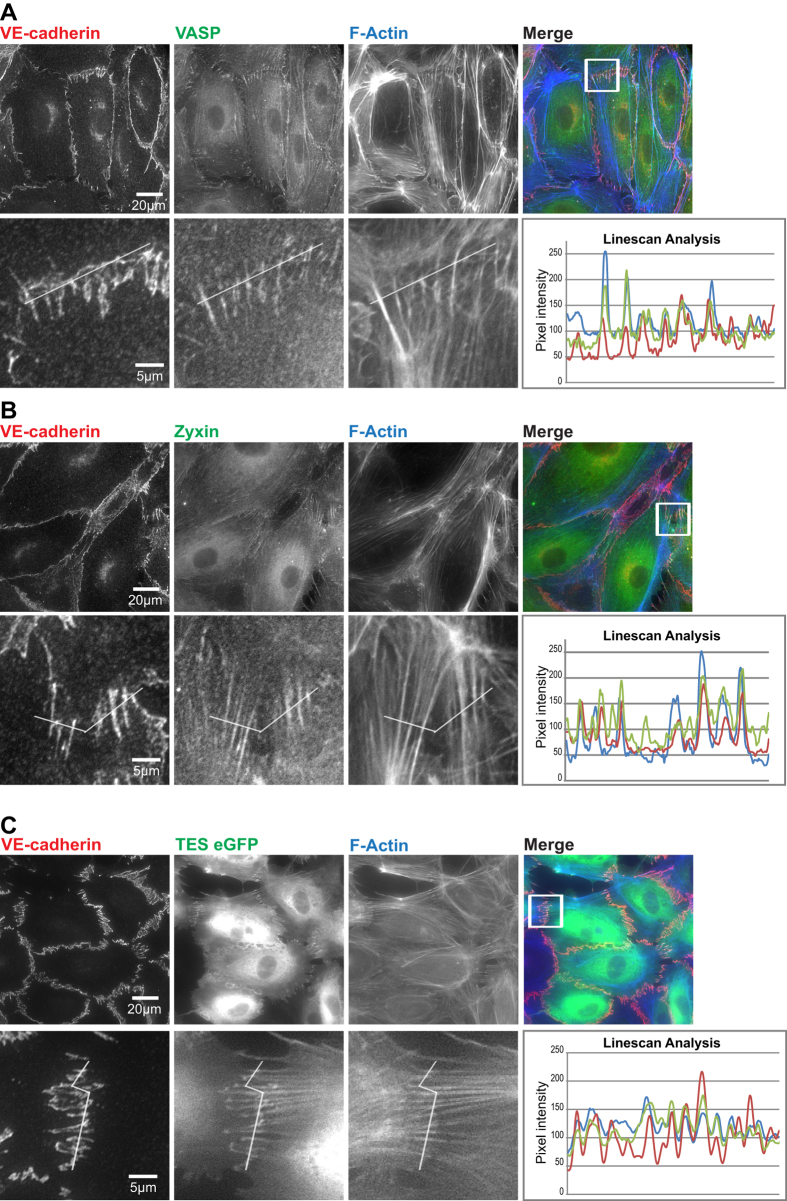
VASP, zyxin and TES concentrate at Focal Adherens Junctions. HUVECs were IF labelled as indicated and subsequently imaged by wide-field microscopy. (**A**–**C**) Bottom row shows a zoom in of a FAJs, indicated by the white square in the top row of images. Line scan analysis of pixel intensity was performed on these FAJs to roughly assess co-localization of the intense signals in the different channels. (**C**) For TES imaging, cells were virally transduced with TES-eGFP before fixation and IF labelling.

VASP and zyxin were previously found to associate with nascent forming cell-cell junctions (which we would now call FAJs) where they may regulate F-actin organization^17^,^19^,^20^. TES is a mutual interactor of these proteins involved in their localization and function at integrin adhesions^25^. We first assessed whether the distribution of these proteins in HUVECs was similar to that of vinculin. Immunofluorescence (IF) stainings show that VASP localizes at VE-cadherin-based junctions and to a subfraction of the F-actin cytoskeleton (Fig. 2A). The intensity of the VASP signal in cell-cell junctions does not follow the intensity profile of the VE-cadherin signal: VASP levels are specifically high in FAJs, which are distinguished by their contact to perpendicular F-actin bundles. The VASP signal is relatively low in LAJs, which are aligned by parallel F-actin. Junctional line scan analysis confirmed that VASP is concentrated in the FAJs, together with VE-cadherin and F-actin. The subcellular localization of zyxin is very similar to that of VASP: The anti-zyxin antibody labelled a subset of F-actin bundles, marked cell-cell junctions faintly in general, but was strongly concentrated in FAJs attached to perpendicular F-actin (Fig. 2B). To investigate the localization of TES, for which no IF-suitable antibodies are available, we lenti-virally expressed TES-eGFP construct^25^. Similar to VASP and zyxin, TES localizes to the FAJs (Fig. 2C). Since VASP, zyxin and TES are F-actin-binding proteins, it is reasonable to expect these proteins at sites of FAJs as well as to other F-actin-rich structures. However the images in Fig. 2 show that VASP, zyxin and TES are much more concentrated with the cadherin-signal at FAJs compared to the surrounding F-actin cytoskeleton. These data thus demonstrate that VASP, zyxin and TES are all enriched specifically in FAJs, suggesting that there are changes in the cadherin complex at FAJs that mediate their recruitment.

### Thrombin induces the recruitment of VASP, zyxin and TES to cell-cell junctions

**Figure 3:**
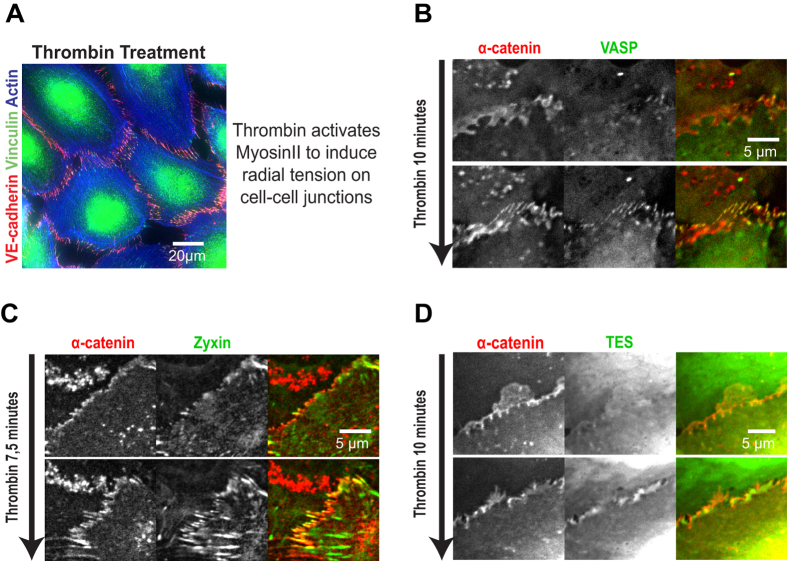
Thrombin treatment recruits VASP, zyxin and TES to cell-cell junctions. (**A**) HUVECs treated with thrombin for 10 minutes (and subsequently fixed and IF labelled for Vinculin, VE-cadherin and Actin) show radial stress fibre formation and presence of vinculin in the associated FAJs, which indicates high tension on these cell-cell junction complexes. (**B**–**D**) HUVECs were virally transduced with expression constructs for α-catenin (tagged by mCherry) as a constitutive cell-cell junction marker and the indicated tension-recruited proteins (tagged by eGFP). Live, wide-field imaging was performed to capture the transition induced by thrombin addition. Still images from the resulting time-lapse series just prior to thrombin treatment and 10 minutes after treatment are shown. Also see Supplemental movie 1–3.

We next investigated whether the recruitment of VASP, zyxin and TES to cell-cell junctions, is induced in the same manner as the junctional recruitment of vinculin. Therefore we used the endothelial permeability factor thrombin that activates RhoA and induces actomyosin contraction in HUVECs^27^. This results in high tension on cell-cell junctions and the robust appearance of FAJs as witnessed by junctional morphology and the strong concentration of vinculin at VE-cadherin-based cell-cell junctions. This is illustrated by Fig. 3A and previously shown in movie 4^9^. To investigate their recruitment dynamics we Lenti-virally expressed VASP-eGFP, zyxin-eGFP or TES-eGFP together with mCherry-tagged α-catenin as a constitutive cadherin complex marker. Live-imaging shows that the formation of FAJs induced by thrombin is immediately followed by a strong increase of VASP, zyxin and TES at these junctions (Fig. 3B–D; Supplemental Movies 1–3). These results demonstrate that the thrombin-induced recruitment of VASP, zyxin and TES to cell-cell junctions closely resembles the mechanosensitive recruitment of vinculin.

### The localization of VASP, zyxin and TES at FAJs is tension-dependent

**Figure 4:**
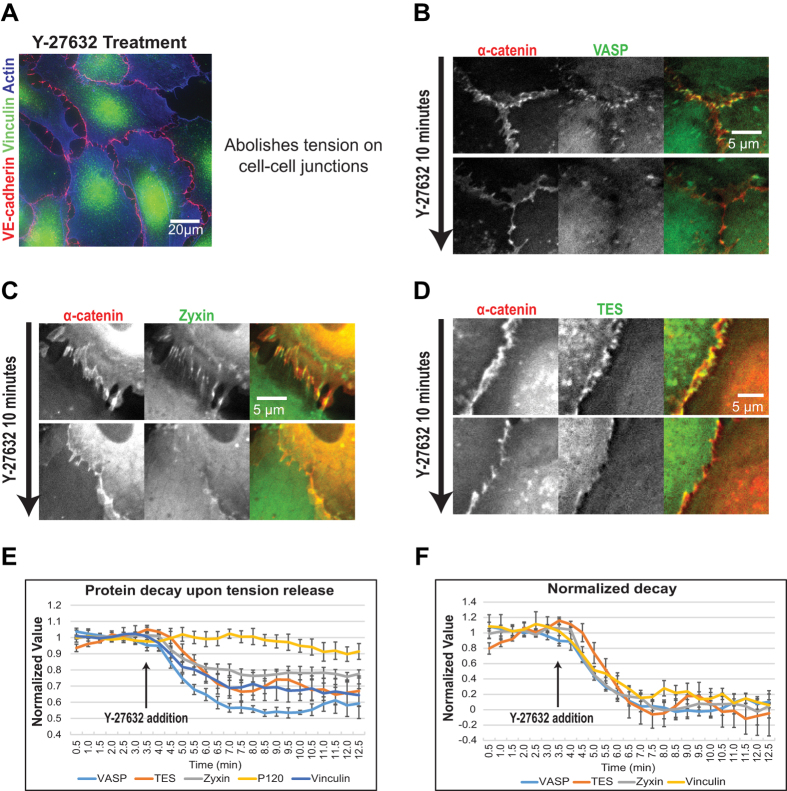
Release of tension removes VASP, zyxin and TES from junctions. (**A**) HUVECs treated with Y-27632 for 10 minutes (and subsequently fixed and IF for vinculin, VE-cadherin and actin) show an absence of radial stress fibres and FAJs (no vinculin in cell-cell junctions), indicating a lack tension on the adherens junctions. (**B**–**D**) HUVECs were virally transduced with expression vectors for α-catenin (tagged by mCherry) as a constitutive junction marker and the indicated tension-regulated proteins (tagged by eGFP) and live, wide-field imaging was performed to capture the result of releasing tension by addition of the Rock inhibitor Y-27632. Still images from these time-lapse series, just prior to Y-27632 treatment and 10 minutes after treatment are shown. Also see Supplemental movies 4-7. (**E**) Pixel intensity of either p120, vinculin, VASP, zyxin or TES was measured over time, in several cell-cell junctions (defined by α-catenin as junctional marker; n ≥ 6) over several time lapse experiments. Pixel intensity before addition of Y-27632 (at 3.5 minutes) was normalized to 1. Vinculin, VASP, zyxin and TES show clear protein decay over time after Y-27632 addition, compared to p120 as control. (**F**) After normalizing the minimum protein intensity to 0, the protein decay of vinculin, VASP, zyxin and TES starts immediately after Y-27632 addition. Furthermore, the protein dynamics of vinculin, VASP, zyxin and TES are similar after Y-27632 addition.

To investigate whether VASP, zyxin and TES localization to cell-cell contacts is tension dependent, we used the Rock inhibitor Y-27632. Addition of Y-27632 results in an almost immediate loss of radial F-actin stress fibers and a concomitant release of tension from cell-cell junctions as witnessed by the absence of vinculin from junctions in Y-27632-treated HUVECs. This is illustrated in Fig. 4A and was previously shown in movie 6^9^. HUVECs were pre-treated for 5 minutes with thrombin to maximize FAJ formation. Subsequently, when Y-27632 was added, we observed a rapid decrease of VASP, zyxin and TES from FAJs amounting to a complete loss within 10 minutes, when junctional intensity did no longer exceed background intensity (Fig. 4B–D; Supplemental Movies 4–6). This demonstrates that the junctional anchor(s) of VASP, zyxin and TES rapidly loses affinity for these proteins or is lost from junctions itself. This junctional anchor could be a mechanosensitive protein, or could be a specific structure of F-actin that is rapidly changing upon inhibition of myosin activity.

To investigate whether the disappearances of VASP, zyxin and TES from cell-cell junctions upon release of tension follow the same or different kinetics among each other, we quantified the decrease of signal intensity over time in cell-cell junctions (n ≥ 6) from multiple time-lapse image series. Cell-cell junctions were distinguished from background by manual tresholding of the α-catenin (mCherry) channel and the average intensity of the junctional pixels was measured in the eGFP channel and divided by the average pixel-intensity of the same pixels in the α-catenin channel to obtain a relative junctional intensity. The relative intensity was normalized to the average value measured before addition of Y-27632. The quantification shows a rapid decrease of VASP, zyxin and TES from cell-cell junctions whereas the ratio between a second core cadherin complex member, p120-catenin, and α-catenin remains stable (Fig. 4E). Normalizing the minimum intensity value reached after full decay (corresponding to the background intensity, which is different for each protein) of every protein to zero shows that disappearance of VASP, zyxin and TES from junctions starts immediately after Y-27632 addition and displays very similar kinetics (Fig. 4F). For reference, vinculin decay upon Y-27632 addition was measured against p120-catenin as a core complex marker in the same experimental settings (see Supplemental Movie 7) and also displays very similar kinetics (as previously quantified in^6^).

Thus our data show that VASP, zyxin and TES localization to cadherin-based junctions is tension-sensitive, with very similar kinetics and in a manner that is very similar to that of Vinculin.

### Mutational analyses reveal that VASP and zyxin form a complex at FAJs

**Figure 5:**
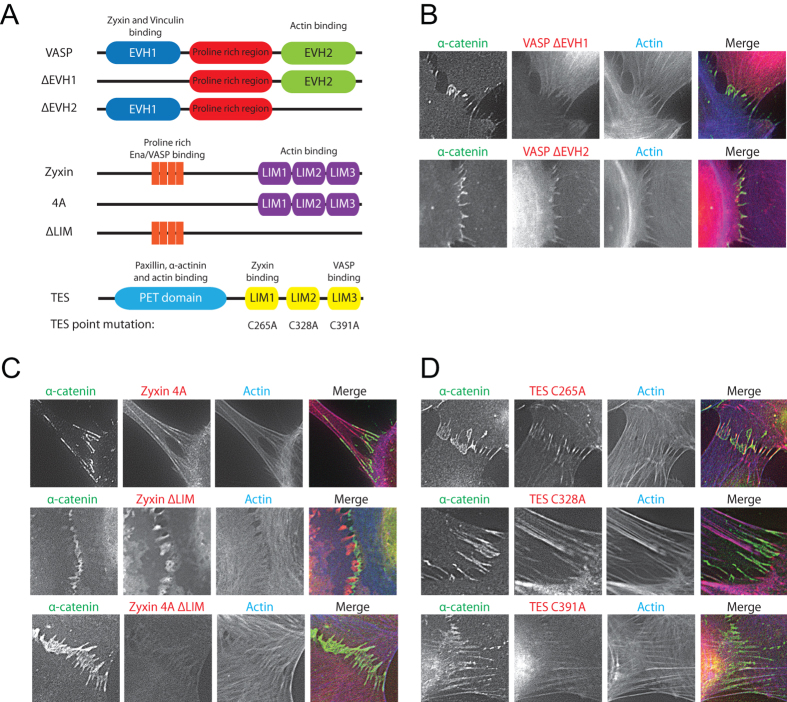
Mutational analysis demonstrates VASP and zyxin are recruited in complex to FAJs. (**A**) Representation of VASP, zyxin and TES mutants used for mutational analysis. (**B**–**D**) Wide-field microscopy images of FAJs in HUVECs transduced with mutant constructs (**B**) HUVECs were lentivirally transduced with eGFP-VASP ΔEVH1 or ΔEVH2 mutant constructs, treated with thrombin for 10 minutes to induce FAJ formation and subsequently fixed and IF labeled for α-catenin and F-actin. (**C**) HUVECs were lentivirally transduced with dsRed-Zyxin 4A, ΔLIM and 4A- ΔLIM mutant constructs, treated with thrombin for 10 minutes to induce FAJ formation and subsequently fixed and IF labeled for α-catenin and F-actin. (**D**) HUVECs were lentivirally transduced with eGFP-TES mutant constructs containing point mutations in each TES LIM domain, treated with thrombin for 10 minutes to induce FAJ formation and subsequently fixed and IF labeled for α-catenin and F-actin.

The similarity in kinetics prompted us to further investigate the manner of recruitment of VASP, zyxin and TES to FAJs by mutating their interaction domains as indicated in Fig. 5A. We lenti-virally expressed these mutants in HUVECs and performed an IF staining on the cells for α-catenin and F-actin. The eGFP-tagged VASP-ΔEVH1 mutant still localized to the actin cytoskeleton but was no longer concentrated at the FAJs (Fig. 5B). The VASP-ΔEVH2 mutant concentrated at FAJs, but showed no recruitment to the actin cytoskeleton (Fig. 5B). Direct F-actin binding is thus not necessary for concentration at FAJs, which underscores the notion that a specific conformation of the cadherin complex mediates VASP recruitment. The EVH1 domain interacts to vinculin and zyxin, but as we show below, the presence of vinculin is not needed for VASP recruitment to FAJs. This indicates that binding to zyxin, or an unidentified partner is essential.

DsRed-tagged Zyxin mutants were kindly provided by Marc Hansen and described previously in^28^. Zyxin 4 A shows localization to the actin cytoskeleton and focal adhesions very similar to WT zyxin, but concentration to FAJs is completely lost in this mutant. This is visible in the IF images and live-imaging frames in Fig. 5C and supplemental movie 8. Deletion of the LIM domains abolishes localization to all F-actin structures, including concentration at FAJs (See Fig. 5C and supplemental movie 8). Patchy structures are visible upon fixation and TX-100 extraction during IF procedures (compare localization in supplemental movie 8 and Fig. 5C) that indicate retention of this mutant at membrane domains. The double mutant (4 A-ΔLIM, Fig. 5C bottom panel) demonstrates no localization to any discernible structure. As for VASP, direct interaction to F-actin does not explain zyxin’s concentration at FAJs. The very specific loss from FAJs of the Zyxin 4 A indicates that it has a unique anchor at cadherin junctions that contains an EVH1 domain. VASP is the only EVH1 domain-containing proteins identified at FAJs so far. In conclusion, VASP and zyxin likely depend on their mutual interaction for FAJ recruitment, which indicates that they localize and function there as a complex.

EGFP-tagged TES constructs in which the essential cysteine residues in each of its LIM domains were mutated were kindly provided by Theresa Higgins and Michael Way and previously characterized in^25^. The images in Fig. 5D show that mutation of the LIM3 domain disrupts the concentration of TES at FAJs, whereas LIM1 seems non-essential and mutation of LIM2 confers strong localization to all F-actin rich cellular structures. TES therefore appears not to depend directly on its interaction with zyxin for localization to FAJs. This indicates a different anchor for TES at cadherin-based FAJs than at integrin-based FAs where its localization does depend on zyxin^25^.

We conclude from these analyses that zyxin and VASP likely localize and function in complex with each other at FAJs, whereas TES may be recruited separately, unlike its zyxin dependent recruitment to integrin-based adhesions. The exact anchors of these proteins at FAJs remain elusive.

### The mechanosensitive localization of VASP, zyxin and TES at FAJs is independent of the α-catenin-vinculin module

**Figure 6:**
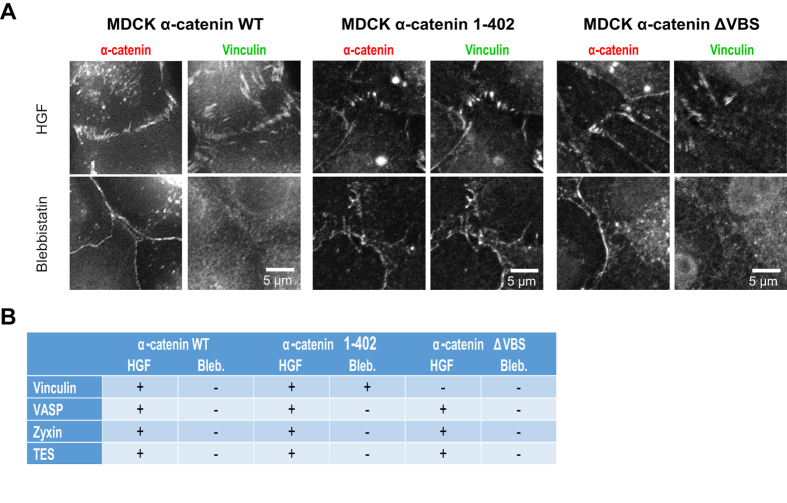
Recruitment of VASP, zyxin and TES to adherens junctions is independent of vinculin. (**A**) Representation of α-catenin wild type and mutants and their effects on vinculin, that were used to investigate whether tension-regulated recruitment of vinculin is necessary for tension-regulated recruitment of VASP, zyxin and TES to the FAJs. (**B**) MDCK cells with constitutive α-catenin knock-down (left panel) were lentivirally transduced with either α-catenin-1-402 (to induce the formation of cell-cell junctions with constitutive, tension insensitive presence of Vinculin; middle panels) or α-catenin-ΔVBS (right panels. Cells were treated with HGF for 2 hrs to maximize tension^30^ or blebbistatin for 30 min. to abolish tension on junctions. (C) Table summarizing the results from the imaging experiments that tested the presence of either vinculin, VASP, zyxin or TES at cell-cell junctions in the different MDCKs cells in the absence or presence of tension. Representative images are shown in figure S1.

To investigate whether the tension-dependent localization of VASP, zyxin and TES to cell-cell junctions is dependent on the α-catenin/vinculin module we investigated junctions in MDCK cells in which we modulated the function of α-catenin. Stable knock-downs of endogenous α-catenin disrupts junction formation in MDCKs^26^. Re-expression of GFP-WT-α-catenin fully rescues junction formation and stability^26^,^29^ and in these cells, vinculin localizes to FAJs after HGF stimulation and is removed from the cell-cell junctions after blebbistatin treatment (Fig. 6A). Expression of either α-catenin-1–402 or α-catenin-ΔVBS also rescues junction formation (Fig 6A), see also^26^ for extensive characterization. α-catenin-1–402 has an intact β-catenin- and vinculin binding domain, but lacks the C-terminal tail that contains the tension-sensitive auto-inhibition domains^10^. It displays constitutive vinculin binding, resulting in the formation of cell-cell junctions in which vinculin is present regardless of the tensile stresses applied at these junctions^10^,^26^. Figure 6A (middle panel) shows that vinculin is equally concentrated at both LAJs and FAJs that are formed by α-catenin-1–402. Expression of the α-catenin-ΔVBS mutant, in which specifically the vinculin binding site is perturbed, results in the formation of phenotypically normal cell-cell junctions, that can appear as FAJs or LAJs and contact radial or parallel F-actin bundles (see also Fig. 1). Regardless of their appearance, these cell-cell junctions are devoid of vinculin, even upon treatment with HGF, which induces actomyosin contractility and tension on cell-cell junctions (Fig 6A, right panel)^26^,^30^. IF stainings of cells expressing α-catenin-402 show that VASP, zyxin and TES are recruited only to the FAJs, even though both LAJs and FAJs contain vinculin (Fig. 6B, and S1A and B). Treatments to inhibit actomyosin contraction by blebbistatin, demonstrate that VASP, zyxin and TES are removed from cell-cell junctions even though vinculin is still present in α-catenin-402-based junctions. Conversely, in cells expressing α-catenin-ΔVBS that were treated with HGF to induce tension and FAJ formation, VASP, zyxin and TES are localized at FAJs, even though vinculin is absent. These results clearly show that the tension-dependent recruitment of VASP, zyxin and TES to cell-cell junctions can occur in the absence of α-catenin stretching or recruitment of vinculin.

### Superresolution microscopy reveals distinct distribution of core-cadherin complex proteins and tension-regulated proteins within FAJs

**Figure 7:**
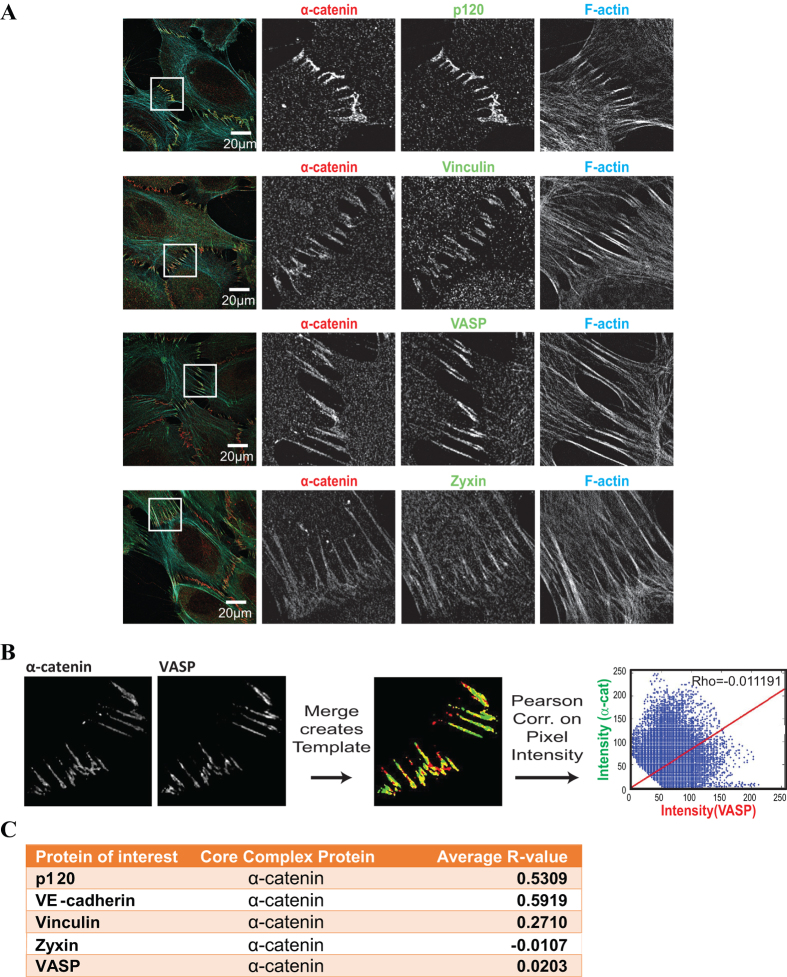
Low correlation of tension-sensitive protein intensity and core-cadherin complex intensity at the ultrastructural level. (**A**) Representative images of Structured Illumination Microscopy performed on HUVECs that were IF labelled for a core cadherin complex protein (α-catenin or VE-cadherin), F-actin and the indicated tension-regulated protein (or a second core member). (**B**) Pearson-correlation analysis of pixel intensities between the core junctional marker and the tension-regulated or second core protein. Pixels included were selected by the generation of a template image comprised of a merge of manually tresholded images from the two separate channels. (**C**) Averages of the Pearson Correlation Coefficient (R) over several images (n = 5) are represented in this table. A Pearson Correlation Coefficient of 1 indicates a perfect correlation between the two proteins, whereas an R of 0 indicates no correlation between two proteins.

The above experiments indicate that multiple mechanosensitive modules exist at the cadherin-F-actin interface. The α-catenin/vinculin module is localized at the core of the cadherin complex. The independence of VASP, zyxin and TES from this module might place these proteins more peripherally, for instance at tension-induced structures in the cadherin proximal F-actin: It has been shown that tension can change F-actin structure to alter the affinity of binding partners including cofilin and MyosinII^31^,^32^. Moreover zyxin has been shown to associate preferentially with damaged or tensile sites within actomyosin bundles^22^,^23^, which could be resembled by the F-actin structure at FAJs. We used Structured Illumination Microscopy (SIM)^33^, in order to distinguish the localization of the different proteins along the cadherin-F-actin axis. HUVECs were fixed and IF labeled for a core cadherin complex protein (α-catenin, p120-catenin or VE-cadherin), for F-actin, and for one of the tension-sensitive proteins VASP, zyxin or vinculin. We did not include TES in these experiments, since a suitable antibody was not available and the use of a GFP-tagged version appeared to increase background in the SIM images and thus affected subsequent analysis and comparison to the other proteins. Representative images showing the full field of view as well as enlarged areas of interest containing FAJs are shown in Fig. 7A. Confirming the results from conventional fluorescence microcopy in Fig. 2, vinculin, VASP and zyxin localize preferentially to the FAJs. However, protein concentration is not homogeneous within FAJs. The SIM data show that cadherin core proteins, tension-sensitive proteins and F-actin do not concentrate at identical locations. A linear order in these concentration profiles is not apparent. It is conceivable that the multiple cadherin complexes within FAJs are not aligned in a way that would allow such a homogenous visualization of the cadherin-F-actin axis.

To further delineate the organization of the FAJ, we quantified the correlation between the intensity distributions of vinculin, VASP and zyxin and the co-stained core-cadherin complex members by Pearson analysis. For this analysis, FAJ-pixels were segmented from background by creating a template image (see methods section) comprised of a merge of the manually tresholded images from the core protein and tension sensitive protein channels (Fig. 7B). The average Pearson correlation coefficient (R) of multiple images (n = 5) for a number of combinations is displayed in Fig. 7C. Our analysis revealed that the correlation between α-catenin with VE-cadherin (R = 0.5919) or p120 (R = 0.5309) was high, as expected for two proteins of the core-cadherin complex. VASP (R = 0.0203) and zyxin (−0.0107) showed no correlation of concentration with the core-cadherin module and vinculin (R = 0.2710) showed intermediate correlation of concentration with the cadherin-module. Thus the superresolution imaging revealed that multiple different protein clusters exist at FAJs, which is consistent with the above findings that multiple mechanosensitive modules exist at the cadherin-F-actin interface.

## Discussion

Previously, we demonstrated that vinculin is involved in cadherin mechanotransduction at a specific sub-set of cadherin-based cell-cell junctions, the Focal Adherens Junctions where it serves to tighten junctions under tension^9^. In this study we show that a number of actin-regulating proteins, VASP, zyxin and TES, are similarly enriched in these FAJs in a tension dependent manner. This similarity suggested a possible co-regulation of VASP, zyxin and TES with vinculin. Co-regulated localization of vinculin and VASP, who are direct interaction partners^34^, has been shown to occur at the ZAJ in columnar epithelial cells^12^. However, our current data show that the localization of these proteins to FAJs is completely independent of vinculin’s presence. Moreover, the use of α-catenin-402 shows that it is also independent of α-catenin’s conformational regulation by mechanical tension.

This suggests that these actin regulators localize to a different, unidentified mechanosensitive module of the FAJ. Each of these proteins contains F-actin binding capacity and they can all interact with each other. Their interaction with F-actin is not ubiquitous, but limited to specific sites within the cytoskeleton. Zyxins interaction with F-actin was shown to be sensitive to myosinII activity and tension^22^ and is recruited to damaged sites in stress fibers^24^. VASP is recruited through zyxin to these sites in F-actin^35^, but also localizes to F-actin structures through TES^25^ or by virtue of its own F-actin binding capacity^36^. Mechanosensitivity of the interaction of TES and F-actin has not been investigated, but TES was shown to be recruited to FAs by zyxin^25^.

In this paper we demonstrate that VASP and zyxin do not need their F-actin-binding domains for FAJ localization. VASP requires its zyxin binding-domain for recruitment to the FAJs, whereas zyxin requires its Ena/VASP binding domain to localize to FAJs. Localization to FAJs specifically, and not to other F-actin structures was maintained in VASP-ΔEVH2 and in Zyxin-ΔLim (Fig. 5) and localization to FAJs specifically was affected in VASP-ΔEVH1 and in Zyxin-4 A. These observations argue that endogenous proteins, through reported intramolecular interactions^25^,^37^,^38^ did not affect localization of the mutants (as this would affect all F-actin-rich structures they localize to). Thus our experiments suggest that VASP and zyxin require binding to each other for recruitment to FAJ. We found no evidence for a direct interaction between TES and zyxin/VASP to be necessary for its tension-sensitive recruitment to FAJs. The tension sensitive concentration of the proteins could be caused by the tension-induced deformation of F-actin fibers, which was shown to change the binding affinity to a number of proteins^39^. Another possible explanation is that the structure of the actin cytoskeleton close to the cadherin junction mimics the structure of damaged stress fibers that was shown to recruit VASP and zyxin. This structure could be myosin sensitive, but independent of tension generated in the associated bundles. Nevertheless, because the previously identified F-actin-binding domains of zyxin and VASP are not essential for their concentration at FAJs, additional unidentified proteins are likely needed to anchor zyxin, VASP and TES to FAJs.

**Figure 8:**
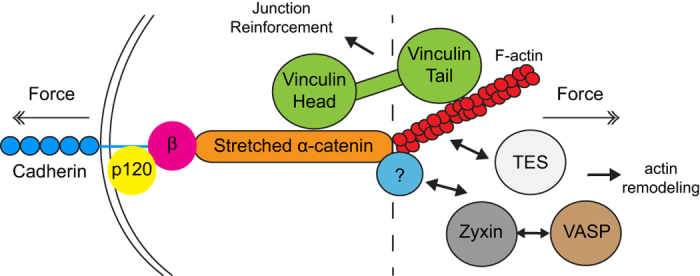
Hypothetical model of tension sensitive localization of VASP, zyxin and TES to the F-actin side of the FAJ. This model represents our current hypothesis of how different FAJs proteins are localized along the cadherin-actin axis. Multiple studies show that vinculin is recruited to α-catenin, regulates junction reinforcement, and is transiently localized to the core-cadherin complex (left of the dotted line). Based on our current data, their F-actin binding capacity, and previous reports about tension-sensitivity we hypothesize that VASP, zyxin and TES are recruited to the actin side of the FAJ, and that this recruitment is independent of the regulation of vinculin.

We summarize the hypothetical linear organization of modules along the cadherin-F-actin axis in Fig. 8. Consistent with this model, super resolution imaging in Fig. 7 reveals that the FAJ consists of hotspots of protein concentration, in which the concentration of members of the core cadherin complex and the tension sensitive proteins does not well correlate. The fact that these hotspots do not align linearly as drawn in Fig. 8 could be explained by the assumption that cadherin-F-actin complexes concentrate in FAJs in a non-aligned fashion. Vinculin concentration correlates intermediately with that of α-catenin, which is consistent with its binding to only the stretched subset of α-catenin or with its interaction with 2 distinct modules within the FAJ (the core cadherin complex and the F-actin content). The complete absence of correlation of concentration of VASP and zyxin with that of the core members could be explained by the hypothesis that these proteins are binding to the F-actin content of the FAJ (Fig. 8).

It is possible that VASP, zyxin and TES cooperate in the force-dependent tightening of cadherin junctions α-catenin and vinculin are involved in^6^,^9^,^26^,^40^. However, an additional function of these proteins at FAJs that extends beyond vinculin is more likely. Vinculin is dispensable for F-actin organization at FAJs (Fig. 1), while one of the functions attributed to zyxin and VASP is to regulate actin organization at sites of adhesion^20^,^28^,^35^. Due to their crucial presence in integrin-based FAs and along the entire actomyosin cytoskeleton, knocking down VASP, zyxin or TES results in overall defects in cellular mechanics (our unpublished observations), which currently precludes proper assessment of their specific function at FAJs. Further establishment of the molecular interactions and their hierarchy in this complex are needed to dissect the mechanisms of recruitment and allow precise perturbations.

In conclusion, our data show that FAJs contain multiple mechanosensitive modules. The α-catenin-vinculin module resides within the force-chain between cadherin and F-actin and regulates adhesion strength. Zyxin, VASP and TES may represent a module that associates to junction proximal actin, of which the structure changes with fluctuating tension, to regulate F-actin organization. Such a multi-layered structure of the FAJ bears similarity to the integrin-based FA and further underscores that the complexity of cadherin-based cell-cell junctions exceeds our current knowledge.

## Material and Methods

### Cells and cell culture

Pooled Human Umbilical Vein Endothelial Cells (HUVECs) from different donors (Lonza) were cultured in EBM-2 medium supplemented with the necessary growth factors (bulletkit; Lonza) on gelatin-coated culture flasks/dishes. Madin-Darby Canine Kidney (MDCK) cells were cultured in Dulbecco’s Modified Eagle Medium (DMEM) supplemented with Fetal Bovine Serum (FBS; 10%) and antibiotics. MDCK cells with α-catenin knock-down, rescued with either α-catenin 1–402 or α-catenin ΔVBS^26^ were cultured together with Geneticin (G418) and Puromycin to maintain knock down and rescue.

### Antibodies and reagents

Mouse mono-clonal antibodies for VE-cadherin and VASP were acquired from BD Biosciences. Zyxin mouse mono-clonal antibodies were purchased from Life Technologies. Rabbit poly-clonal Vinculin antibodies were used for Figs 1, 3 and 4, Vinculin (hVin-1 clone) mouse mono-clonal antibodies were used for Figs 6 and 7, and both were obtained from Sigma-Aldrich. Rabbit poly-clonal antibodies for α-catenin were also purchased from Sigma-Aldrich. Alexa488 and Alexa594 secondary antibodies were purchased from Life Technologies and Phalloidin415 was obtained from Promokine. Y-27632 was used at 10 μM, blebbistatin was used at 100 μM and both were acquired from EMD Millipore. Human plasma-derived thrombin (used at 0,2 μg/ml), calf skin derived Collagen (coating coverslip with 30 μg/ml) and Fibronectin (coating coverslips and Lab-Tek chambers with 3 μg/ml) were purchased from Sigma-Aldrich. Recombinant human HGF was purchased from R&D systems and used 500 ng/ml.

### Lentiviral transduction and constructs

In order to acquire lentiviral particles, HEK293 cells were transiently transfected with both third-generation packaging constructs and the lentiviral expression vectors, and supernatant containing the lentiviral particles was isolated 2–3 days post transfection. HUVECs and MDCKs were transduced with the lentiviral particles overnight together with 8 μg/ml polybrene. pLV-CMV vectors containing mCherry-α-catenin, eGFP-Vinvulin, eGFP-VASP and Zyxin-eGFP were used in Figs 3 and 4. For IF fixed imaging, cells were transduced with TES, since no IF applicable antibody is available. pLV eGFP-TES (Figs 2, 3 and 4), RFP-TES (Fig. 6) and the eGFP TES mutants (Fig. 5) were kindly provided by Theresa Higgins and Michael Way. The mutant zyxin constructs were kindly provided by Marc Hansen and were fluorescently tagged with dsRED. For live investigation of the zyxin mutants we used the epithelial cell line DU145. To create the VASP mutants used for Fig. 5, we performed a PCR deletion on the pEGFP-C2-VASP template construct. To create VASP ΔEVH1 we used a forward primer (CAAGCTTCGAATTCATGCTGGAACAACAGAAAAG) and a reverse primer (CTTTTCTGTTGTTCCAGCATGAATTCGAAGCTTG), to establish a deletion in the EVH1 domain of the template construct. To create VASP ΔEVH2 we used a forward primer (CAATAGTGGGGGTTCCTTGACTCGACGGTAC) and a reverse primer (GTACCGTCGAGTCAAGGAACCCCCACTATTG), to establish a deletion in the ΔEVH2 domain of the template construct.

### IF and live wide-field microscopy

For IF stainings, cells were plated on coverslips coated with either Fibronectin (HUVECs) or Collagen (MDCKs). Prior to fixation with 2% paraformaldehyde for 20 minutes, MDCK cells were treated with either HGF or blebbistatin (Fig. 6 and figure S1). After fixation, cells were permeabilized with 0,4% Triton X-100 for 5 minutes and blocked in 2% BSA for 1 hour. Phalloidin, primary- and secondary antibodies were diluted in 2% BSA and incubated with the cells for 1 hour. Afterwards, cells were mounted in Mowiol 4–88/DABCO solution (Sigma-Aldrich). For live imaging, lentivirally transduced HUVECs were plated into Lab-Tek chambered 1.0 boro-silicate coverglass slides coated with fibronectin and cultured in EBM-2 medium supplemented with EGM-2 bulletkit. Live (at 37 °C) and fixed cells were imaged using an inverted research widefield microscope (Eclipse Ti; Nikon) with perfect focus system, equipped with a 60 × 1.49 NA Apochromat total internal reflection fluorescence (oil) objective lens, a microscope cage incubator (OkoLab), and an EM charge-coupled device (CCD) camera (Andor Technology) controlled with NIS-Elements Ar 4.0 software.

### Structured Illumination Microscopy

Imaging was performed using a Zeiss Elyra PS1 system. 3D-SIM data was acquired using a 63 × 1.4NA oil objective. 488, 561, 642 100 mW diode lasers were used to excite the fluorophores together with respectively a BP 495–575 + LP 750, BP 570–650 + LP 75 or LP 655 excitation filter. For 3D-SIM imaging a grating was present in the light path. The grating was modulated in 5 phases and 5 rotations, and multiple z-slices with an interval of 110 nm were recorded on an Andor iXon DU 885, 1002 × 1004 EMCCD camera. Raw images were reconstructed using the Zeiss Zen software.

### Image Analysis

Any image adjustments in this report were performed with ImageJ software (developed by the NIH). For Figs 1, 2, 3, 4, 5, images were treated with an unsharp mask (3.0/0.6), and brightness/contrast was adjusted. Line scan analysis for Figs 1 and 2 was performed with ImageJ, furthermore, the data in Fig. 1 was normalized to average pixel intensity and plotted in a graph using a running average. For the live imaging (supplemental movies 1–8) and the representative still images (Figs 3 and 4) unsharp mask (3.0/0.6) was used, brightness/contrast was adjusted, and a subtract background and/or Gaussian blur was performed where appropriate. For calculating the protein decay at cell-cell junctions treated with Y-27632 (Fig. 4E,F), both α-catenin movies and Vinculin, VASP, zyxin, TES and p120 movies (n ≥ 6) were treated with a subtract background of 20.0 and brightness/contrast was adjusted for visibility. These α-catenin movies were then treated with an unsharp mask (3.0/0.6) and a Gaussian blur (1.0), afterwards a threshold was added to distinguish cell-cell junctions from background. The thresholded movies were used as template for measuring the average pixel intensity in both α-catenin and Vinculin, VASP, zyxin, TES, p120 channel. Average pixel intensity for Vinculin, VASP, zyxin, TES and p120 was divided by average pixel intensity for α-catenin, to obtain the relative junctional intensity. For Fig. 4E, the relative intensity of Vinculin, VASP, zyxin, TES and p120 before Y-27632 addition was normalized to 1. For Fig. 4F, the minimum relative intensity of Vinculin/VASP/zyxin/TES was also normalized to 0. Representative images in Fig. 6 were only adjusted for brightness/contrast. For analysis of the SIM superresolution images, still images of the core-cadherin complex- and tension-regulated proteins were treated with a Gaussian blur of 2.0. A merge of certain proteins (Fig. 7C) was performed, treated with a Gaussian blur of 1.0 and thresholded (Fig. 7B) and used as template image. Average (n = 5) Pearson correlation coefficients were calculated using MATLAB (Mathworks) by correlating the pixel intensities of the protein of interest and core-complex protein localized in the template images (Fig. 7C).

## Additional Information

**How to cite this article**: Oldenburg, J. *et al.* VASP, zyxin and TES are tension-dependent members of Focal Adherens Junctions independent of the α-catenin-vinculin module. *Sci. Rep.* **5**, 17225; doi: 10.1038/srep17225 (2015).

## Supplementary Material

Supplementary Information

Supplemental Movie 1

Supplemental Movie 2

Supplemental Movie 3

Supplemental Movie 4

Supplemental Movie 5

Supplemental Movie 6

Supplemental Movie 7

Supplemental Movie 8

## References

[b1] Galbraith C. G., Yamada K. M. & Sheetz M. P. The relationship between force and focal complex development. The Journal of cell biology 159, 695–705, 10.1083/jcb.200204153 (2002). 12446745 PMC2173098

[b2] Roca-Cusachs P. *et al.* Integrin-dependent force transmission to the extracellular matrix by alpha-actinin triggers adhesion maturation. Proceedings of the National Academy of Sciences of the United States of America 110, E1361–1370, 10.1073/pnas.1220723110 (2013). 23515331 PMC3625291

[b3] Rehfeldt F. *et al.* Hyaluronic acid matrices show matrix stiffness in 2D and 3D dictates cytoskeletal order and myosin-II phosphorylation within stem cells. Integrative biology: quantitative biosciences from nano to macro 4, 422–430, 10.1039/c2ib00150k (2012). 22344328

[b4] Alexander N. R. *et al.* Extracellular matrix rigidity promotes invadopodia activity. Current biology : CB 18, 1295–1299, 10.1016/j.cub.2008.07.090 (2008). 18718759 PMC2555969

[b5] Engler A. J., Sen S., Sweeney H. L. & Discher D. E. Matrix elasticity directs stem cell lineage specification. Cell 126, 677–689, 10.1016/j.cell.2006.06.044 (2006). 16923388

[b6] le Duc Q. *et al.* Vinculin potentiates E-cadherin mechanosensing and is recruited to actin-anchored sites within adherens junctions in a myosin II-dependent manner. The Journal of cell biology 189, 1107–1115, 10.1083/jcb.201001149 (2010). 20584916 PMC2894457

[b7] Ladoux B. *et al.* Strength dependence of cadherin-mediated adhesions. Biophysical journal 98, 534–542, 10.1016/j.bpj.2009.10.044 (2010). 20159149 PMC2820642

[b8] Liu Z. *et al.* Mechanical tugging force regulates the size of cell-cell junctions. Proceedings of the National Academy of Sciences of the United States of America 107, 9944–9949, 10.1073/pnas.0914547107 (2010). 20463286 PMC2890446

[b9] Huveneers S. *et al.* Vinculin associates with endothelial VE-cadherin junctions to control force-dependent remodeling. The Journal of cell biology 196, 641–652, 10.1083/jcb.201108120 (2012). 22391038 PMC3307691

[b10] Yonemura S., Wada Y., Watanabe T., Nagafuchi A. & Shibata M. alpha-Catenin as a tension transducer that induces adherens junction development. Nature cell biology 12, 533–542, 10.1038/ncb2055 (2010). 20453849

[b11] Yao M. *et al.* Force-dependent conformational switch of alpha-catenin controls vinculin binding. Nature communications 5, 4525, 10.1038/ncomms5525 (2014). 25077739

[b12] Leerberg J. M. *et al.* Tension-sensitive actin assembly supports contractility at the epithelial zonula adherens. Current biology : CB 24, 1689–1699, 10.1016/j.cub.2014.06.028 (2014). 25065757 PMC5103636

[b13] Huveneers S. & de Rooij J. Mechanosensitive systems at the cadherin-F-actin interface. Journal of cell science 126, 403–413, 10.1242/jcs.109447 (2013). 23524998

[b14] Barzik M., McClain L. M., Gupton S. L. & Gertler F. B. Ena/VASP regulates mDia2-initiated filopodial length, dynamics, and function. Molecular biology of the cell 25, 2604–2619, 10.1091/mbc.E14-02-0712 (2014). 24989797 PMC4148250

[b15] Winkelman J. D., Bilancia C. G., Peifer M. & Kovar D. R. Ena/VASP Enabled is a highly processive actin polymerase tailored to self-assemble parallel-bundled F-actin networks with Fascin. Proceedings of the National Academy of Sciences of the United States of America 111, 4121–4126, 10.1073/pnas.1322093111 (2014). 24591594 PMC3964058

[b16] Breitsprecher D. *et al.* Molecular mechanism of Ena/VASP-mediated actin-filament elongation. EMBO J 30, 456–467, 10.1038/emboj.2010.348 (2011). 21217643 PMC3034019

[b17] Vasioukhin V., Bauer C., Yin M. & Fuchs E. Directed actin polymerization is the driving force for epithelial cell-cell adhesion. Cell 100, 209–219 (2000). 10660044 10.1016/s0092-8674(00)81559-7

[b18] Yoshigi M., Hoffman L. M., Jensen C. C., Yost H. J. & Beckerle M. C. Mechanical force mobilizes zyxin from focal adhesions to actin filaments and regulates cytoskeletal reinforcement. The Journal of cell biology 171, 209–215, 10.1083/jcb.200505018 (2005). 16247023 PMC2171187

[b19] Sperry R. B. *et al.* Zyxin controls migration in epithelial-mesenchymal transition by mediating actin-membrane linkages at cell-cell junctions. Journal of cellular physiology 222, 612–624, 10.1002/jcp.21977 (2010). 19927303

[b20] Nguyen T. N., Uemura A., Shih W. & Yamada S. Zyxin-mediated actin assembly is required for efficient wound closure. The Journal of biological chemistry 285, 35439–35445, 10.1074/jbc.M110.119487 (2010). 20801875 PMC2975167

[b21] Hoffman L. M. *et al.* Genetic ablation of zyxin causes Mena/VASP mislocalization, increased motility, and deficits in actin remodeling. The Journal of cell biology 172, 771–782, 10.1083/jcb.200512115 (2006). 16505170 PMC2063708

[b22] Uemura A., Nguyen T. N., Steele A. N. & Yamada S. The LIM domain of zyxin is sufficient for force-induced accumulation of zyxin during cell migration. Biophysical journal 101, 1069–1075, 10.1016/j.bpj.2011.08.001 (2011). 21889443 PMC3164133

[b23] Smith M. A. *et al.* LIM domains target actin regulators paxillin and zyxin to sites of stress fiber strain. PloS one 8, e69378, 10.1371/journal.pone.0069378 (2013). 23990882 PMC3749209

[b24] Smith M. A. *et al.* A zyxin-mediated mechanism for actin stress fiber maintenance and repair. Developmental cell 19, 365–376, 10.1016/j.devcel.2010.08.008 (2010). 20833360 PMC2954498

[b25] Garvalov B. K. *et al.* The conformational state of Tes regulates its zyxin-dependent recruitment to focal adhesions. The Journal of cell biology 161, 33–39, 10.1083/jcb.200211015 (2003). 12695497 PMC2172870

[b26] Twiss F. *et al.* Vinculin-dependent Cadherin mechanosensing regulates efficient epithelial barrier formation. Biology open 1, 1128–1140, 10.1242/bio.20122428 (2012). 23213393 PMC3507192

[b27] van Nieuw Amerongen G. P., Musters R. J., Eringa E. C., Sipkema P. & van Hinsbergh V. W. Thrombin-induced endothelial barrier disruption in intact microvessels: role of RhoA/Rho kinase-myosin phosphatase axis. American journal of physiology. Cell physiology 294, C1234–1241, 10.1152/ajpcell.00551.2007 (2008). 18353893

[b28] Hansen M. D. & Beckerle M. C. Opposing roles of zyxin/LPP ACTA repeats and the LIM domain region in cell-cell adhesion. The Journal of biological chemistry 281, 16178–16188, 10.1074/jbc.M512771200 (2006). 16613855

[b29] Loerke D. *et al.* Quantitative imaging of epithelial cell scattering identifies specific inhibitors of cell motility and cell-cell dissociation. Sci Signal 5, rs5, 10.1126/scisignal.2002677 (2012). PMC5588157 22763340

[b30] de Rooij J., Kerstens A., Danuser G., Schwartz M. A. & Waterman-Storer C. M. Integrin-dependent actomyosin contraction regulates epithelial cell scattering. The Journal of cell biology 171, 153–164, 10.1083/jcb.200506152 (2005). 16216928 PMC2171213

[b31] Hayakawa K., Tatsumi H. & Sokabe M. Actin filaments function as a tension sensor by tension-dependent binding of cofilin to the filament. The Journal of cell biology 195, 721–727, 10.1083/jcb.201102039 (2011). 22123860 PMC3257564

[b32] Uyeda T. Q., Iwadate Y., Umeki N., Nagasaki A. & Yumura S. Stretching actin filaments within cells enhances their affinity for the myosin II motor domain. PloS one 6, e26200, 10.1371/journal.pone.0026200 (2011). 22022566 PMC3192770

[b33] Gustafsson M. G. Surpassing the lateral resolution limit by a factor of two using structured illumination microscopy. Journal of microscopy 198, 82–87 (2000). 10810003 10.1046/j.1365-2818.2000.00710.x

[b34] Huttelmaier S. *et al.* The interaction of the cell-contact proteins VASP and vinculin is regulated by phosphatidylinositol-4,5-bisphosphate. Current biology : CB 8, 479–488 (1998). 9560340 10.1016/s0960-9822(98)70199-x

[b35] Grange J., Moody J. D., Ascione M. P. & Hansen M. D. Zyxin-VASP interactions alter actin regulatory activity in zyxin-VASP complexes. Cellular & molecular biology letters 18, 1–10, 10.2478/s11658-012-0035-2 (2013). 23076992 PMC6275665

[b36] Walders-Harbeck B., Khaitlina S. Y., Hinssen H., Jockusch B. M. & Illenberger S. The vasodilator-stimulated phosphoprotein promotes actin polymerisation through direct binding to monomeric actin. FEBS letters 529, 275–280 (2002). 12372613 10.1016/s0014-5793(02)03356-2

[b37] Kuhnel K. *et al.* The VASP tetramerization domain is a right-handed coiled coil based on a 15-residue repeat. Proceedings of the National Academy of Sciences of the United States of America 101, 17027–17032, 10.1073/pnas.0403069101 (2004). 15569942 PMC535362

[b38] Call G. S. *et al.* Zyxin phosphorylation at serine 142 modulates the zyxin head-tail interaction to alter cell-cell adhesion. Biochem Biophys Res Commun 404, 780–784, 10.1016/j.bbrc.2010.12.058 (2011). 21168386

[b39] Leckband D. E. & de Rooij J. Cadherin Adhesion and Mechanotransduction. Annual review of cell and developmental biology, 10.1146/annurev-cellbio-100913-013212 (2014). 25062360

[b40] Barry A. K. *et al.* alpha-catenin cytomechanics–role in cadherin-dependent adhesion and mechanotransduction. Journal of cell science 127, 1779–1791, 10.1242/jcs.139014 (2014). 24522187 PMC3986676

