# Supplementary material for: VASP, zyxin and TES are tension-dependent members of Focal Adherens Junctions independent of the α-catenin-vinculin module
Source: Sci Rep. 2015 Nov 27;5:17225. doi: 10.1038/srep17225 (PMC4661603; doi:10.1038/srep17225)
Supplement: Supplementary Information [file srep17225-s1.pdf]

VASP, zyxin and TES are tension-dependent members of Focal Adherens Junctions independent of the α-catenin-vinculin module.

Joppe Oldenburg[1], Gerard van der Krogt[1], Floor Twiss[1], Annika Bongaarts[1], Yasmin Habani[1], Johan A. Slotman[2], Adriaan Houtsmuller[2], Stephan Huveneers[3], Johan de Rooij[1]*

[1]Dept. Molecular Cancer Research, Center for Molecular Medicine, University Medical Center Utrecht, Stratenum 3.231, Universiteitsweg 100, 3584 CG, Utrecht, the Netherlands

[2]Erasmus Optical Imaging Center, Department of Pathology, Erasmus MC, Dr. Molewaterplein 50-60, 3015 GE Rotterdam, the Netherlands

[3]Department of Molecular Cell Biology, Sanquin Research and Swammerdam Institute for Life Sciences, University of Amsterdam. Plesmanlaan 125, 1066 CX, Amsterdam, the Netherlands

*Corresponding author:

Dept. Molecular Cancer Research, Center for Molecular Medicine, University Medical Center Utrecht, Stratenum 3.231, Universiteitsweg 100, 3584 CG, Utrecht, the Netherlands.

j.derooij-4@umcutrecht.nl

+31-88-7568961

Running title: Multiple mechanosensitive modules at FAJs

Supplemental Figure/Movie Legends

Figure S1. VASP, zyxin and TES localize to the cell-cell junctions independent of vinculin absence or presence. α-catenin deficient MDCK cells were transduced with either α-catenin 1-402 (recruiting vinculin to cell-cell junctions independently) or α-catenin ΔVBS (no vinculin recruitment to cell-cell junctions) mutants fluorescently tagged with eGFP. (A) α-catenin 1-402 MDCK cells were IF labeled for VASP, zyxin or transduced with TES-RFP. Prior to fixation cell were treated with either HGF (inducing FAJs formation) or blebbistatin (abolishing actomyosin contractility). VASP, zyxin and TES localized to the cell-cell junctions after HGF, but were absent in cell-cell adhesions in blebbistatin treated cells despite continued presence of vinculin. (B) α-catenin ΔVBS MDCK cells were IF labeled for VASP, zyxin or transduced with TES-RFP. Even though vinculin is absent from the junctions, VASP, zyxin and TES still localize tension-dependent to cell-cell adhesions.

Supplemental Movie 1-3.Thrombin induced tension on cell-cell adhesions recruits VASP, zyxin and TES to FAJs. HUVECs were transduced with α-catenin (mCherry) and either VASP, zyxin or TES (eGFP), and treated with thrombin (at 00:00) during live imaging. (1) Live investigation of VASP demonstrates a strong increase of VASP localization at remodeling junctions (FAJs) after thrombin addition (2) Similar to VASP, after thrombin addition, FAJs demonstrate an increase in zyxin localization (3) After thrombin addition, TES is recruited to the FAJs. The pattern of localization of TES is diffuse, however the immediate effect of thrombin on TES localization to FAJs is similar to VASP and zyxin.

Supplemental Movie 4-7. Abolishment of tension by Y-27632 decreases localization of VASP, zyxin, TES and vinculin at cell-cell adhesions. HUVECs were transduced with α-catenin (mCherry) and either VASP, zyxin, TES or vinvulin (eGFP), and treated with Y-27632 (at 00:00) during live imaging. (4) Cell-cell adhesions containing VASP demonstrate a rapid decrease in VASP localization after Y-27632 addition. (5) Zyxin localized at the cell-cell junctions is quickly removed after addition of Y-27632 (6) TES localization at cell-cell junctions

is decreasing rapidly after Y-27632 addition. (7) Cell-cell adhesions containing vinculin demonstrate a rapid decrease in vinculin localization after Y-27632 addition.

Supplemental Movie 8. Altered localization patterns of zyxin mutant proteins. DU145 cells were transduced with either zyxin WT, zyxin 4A, zyxin ΔLIM or zyxin 4A- ΔLIM (dsRED) and subsequently imaged using live microscopy. Whereas wild type zyxin localizes normally to FAJ structures, the mutant proteins do not localize to the cell-cell junctions.

Figure S1

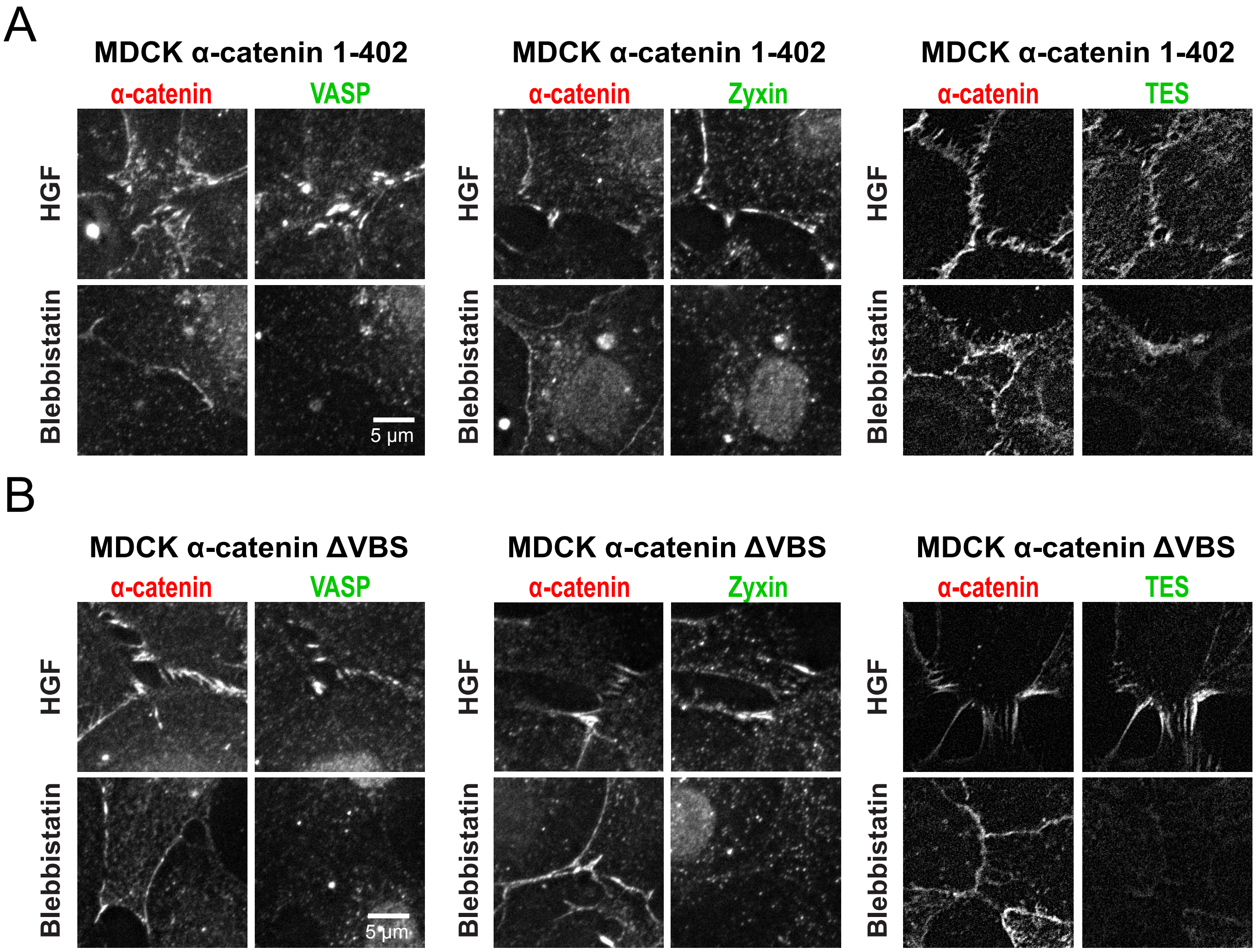
A
MDCK α-catenin 1-402
α-catenin
VASP
HGF
Blebbistatin
5 µm
MDCK α-catenin 1-402
α-catenin
Zyxin
HGF
Blebbistatin
MDCK α-catenin 1-402
α-catenin
TES
HGF
Blebbistatin
B
MDCK α-catenin ΔVBS
α-catenin
VASP
HGF
Blebbistatin
5 µm
MDCK α-catenin ΔVBS
α-catenin
Zyxin
HGF
Blebbistatin
MDCK α-catenin ΔVBS
α-catenin
TES
HGF
Blebbistatin
